# Antioxidant Activity of Silica-Based Bioactive Glasses

**DOI:** 10.1021/acsbiomaterials.1c00048

**Published:** 2021-04-27

**Authors:** Sara Ferraris, Ingrid Corazzari, Francesco Turci, Andrea Cochis, Lia Rimondini, Enrica Vernè

**Affiliations:** † Politecnico di Torino, Department of Applied Science and Technology, Institute of Materials Physics and Engineering, Torino 10129, Italy; ‡ Department of Chemistry and “G. Scansetti” Interdepartmental Center for Studies on Asbestos and Other Toxic Particulates, University of Torino, Torino 10125, Italy; § Department of Health Sciences, Center for Translational Research on Autoimmune and Allergic Diseases−CAAD, University of Piemonte Orientale UPO, Novara 28100, Italy

**Keywords:** bioactive glass, antioxidant, radical scavenging, hydroxyl groups

## Abstract

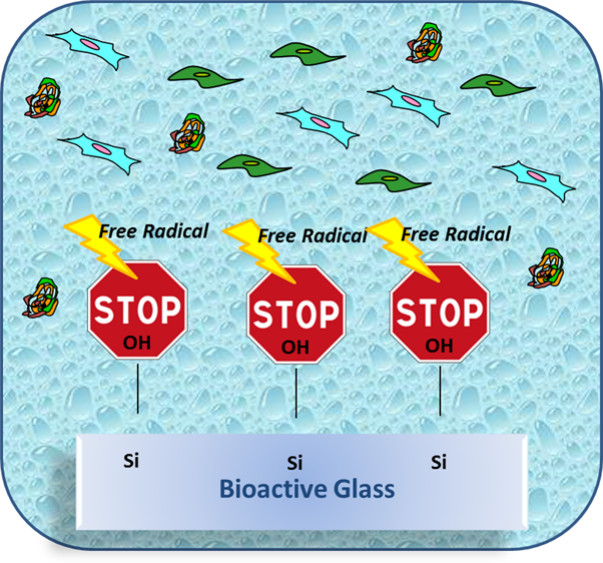

Bioactive glasses are the materials of choice in the field of bone regeneration. Antioxidant properties of interest to limit inflammation and foreign body reactions have been conferred to bioactive glasses by the addition of appropriate ions (such as Ce or Sr). On the other hand, the antioxidant activity of bioactive glasses without specific ion/molecular doping has been occasionally cited in the literature but never investigated in depth. In the present study, three silica-based bioactive glasses have been developed and characterized for their surface properties (wettability, zeta potential, chemical composition, and reactivity) and radical scavenging activity in the presence/absence of cells. For the first time, the antioxidant activity of simple silica-based (SiO_2_–CaO–Na_2_O) bioactive glasses has been demonstrated.

## Introduction

Bioactive glasses have been widely studied and applied mainly in the bone regeneration field due to their ability to bind to the bone and effectively stimulate its regeneration and healing by releasing specific ions, and some applications in contact with soft tissues have also been mentioned.^1^ Their bioactivity mechanism has been widely reported in the literature since the early 1990s as a complex sequence of reactions that takes place on immersion in a simulated body fluid^2,3^ (SBF, a solution with a composition similar to the inorganic fraction of human plasma) and starts with a fast ion exchange between the alkaline ions from the glass surface and the hydrogen ions from the solution followed by the formation, at the surface, of free −OH groups (silanols) that go through polycondensation developing a silica gel layer. This layer stimulates the adsorption of Ca^++^ and PO_4_^3–^ ions from the solution and their reaction to induce hydroxyapatite deposition. Furthermore, silanols with a specific spatial intersilanol distance have been recently proved to be the active site of interaction between the silica surface and the phosphate-charged groups of phospholipids.^4^ Bioactive glasses can be further doped with numerous ions in order to induce specific properties such as osteogenic, antibacterial, or angiogenetic activities. Particularly, recent advances have been reported in formulating bioactive glasses doped with trace and therapeutic elements (e.g., Mg, Zn, Sr, Ag, and Cu) and investigations on the effect of these elements on their biological performance have been reported.^1^

It is also well known that the bone remodeling process is regulated by a variety of biological agents, including, among others, species responsible for local and systemic stress actions.^1^ In particular, cell-derived reactive oxygen species (ROS), such as hydroxyl radical (HO·), superoxide (O_2_^–^), and hydrogen peroxide (H_2_O_2_), are involved in cell-signaling and other physiological functions essential to maintain the homeostasis of cells and tissues. Nevertheless, an excessive production of ROS can damage membrane lipids, proteins, and DNA.^5^ Silica is known to induce cell death from ROS due to the opening of strained surface three-membered Si–O–Si rings.^6^ The imbalance between overproduction of ROS and the consumption of antioxidant cell defenses is related to oxidative stress, which can hinder osteoblast differentiation and mineralization, thus enhancing the osteoclast activity and leading to bone loss and osteoporosis.^7,8^ Several events connected with bone surgery such as trauma, tissue injuries, inflammation, and infection can increase the ROS level. In this context, materials intended for bone contact applications with an antioxidant activity appear extremely promising.

Due to their versatility, the antioxidant capability of bioglasses can be modulated by adding appropriate metal ions. For example, bioactive glasses containing cerium have shown antioxidant activities, like the ability to degrade H_2_O_2_ by mimicking the catalase enzyme.^9−12^ Moreover, a bioactive glass containing strontium demonstrated antioxidant properties by increasing the activity of cellular antioxidant enzymes^13,14^ in addition to the Sr bone stimulation ability. In addition to these specific effects related to the nature of the metal ions added as minor components, an intrinsic antioxidant activity of the main component (i.e., silica) of bioactive glasses has been detected only in very few studies, and it is associated, in first approximation, with their hydroxylation degree,^15,16^ even though further studies have been suggested to support this hypothesis. Moreover, the antioxidant activity has also been reported for silica hydride encapsulated in a silicate substrate.^17−19^

Bioactive glasses with an intrinsic antioxidant activity have high potential in a wide range of biomedical applications. For this reason, in the present research study, three silica-based bioactive glasses containing only SiO_2_, CaO, and Na_2_O in different proportions, but free from specific “antioxidant ions,” have been designed and synthesized. Their surface properties (e.g., wettability, charge, and hydroxylation) and antioxidant ability (in the presence and absence of osteoblast progenitor cells) have been investigated and correlated, for the first time, with their degree of hydroxylation.

## Material and Methods

Three silica-based bioactive glasses with the following molar compositions SCN 50-35-15 (50% SiO_2_, 35%CaO, and 15% Na_2_O), SCN 55-35-10 (55% SiO_2_, 35%CaO, and 10% Na_2_O), and SCN 60-35-5 (60% SiO_2_, 35%CaO, and 5% Na_2_O) were produced via the melt and quenching route in bulk and powder forms. In brief, the precursors (SiO_2_, Na_2_CO_3_, and CaCO_3_) were melted at 1600 °C in a platinum crucible and then poured in water to obtain a frit or on a brass plate to obtain a bar. Glass bars were annealed for 12 h at 550 °C (SCN-35-15), 600 °C (SCN 55-35-10), or 650 °C (SCN 60-35-5) and then cut into 2 mm thick slices using an automatic cutting machine equipped with a diamond blade and were polished with SiC abrasive papers (up to 4000 grit). The glass frit was milled and sieved up to an average size of less than 20 μm. The surface wettability was determined using the sessile drop method (DSA-100, KRÜSS GmbH, Hamburg, Germany) with ultrapure water (5 μL drop) on bulk samples. The zeta potential as a function of pH was analyzed by means of electrokinetic measurements (SurPASS, Anton Paar, with an adjustable gap cell) in 0.001 M KCl titrated with 0.05 M HCl or 0.05 M NaOH.^20^ The surface chemical composition and the chemical state of elements were investigated by X-ray photoelectron spectroscopy (XPS, PHI 5000 VersaProbe, Physical Electronics). Survey spectra (0–1200 eV range) and high-resolution analyses of C, O, and Si using the hydrocarbon C 1s peak at 284.80 eV as a reference signal (charge compensation effect) were collected. Surface reactivity and bioactivity (i.e., the ability to induce the precipitation of hydroxyapatite in vitro) were studied by immersion in an SBF prepared according to Kokubo’s protocol^21^ on powder samples. Glass powder (100 mg, <20 μm) was introduced in a plastic container filled with 25 mL of SBF and was stored at 37 °C for up to 3 days. Fourier transform infrared spectroscopy analyses (FTIR, Alpha, Bruker Optics, Ettlingen, Germany) were performed on glass KBr pellets in the absorption mode before and after SBF soaking. The ROS scavenging activity in inorganic cell-free media was assessed by electron paramagnetic resonance (EPR)/spin-trapping technique employing 5,5-dimethyl-1-pyrroline-1-oxide (DMPO, Alexis Biochemicals, San Diego, CA, USA) as the spin-trapping molecule on buffered suspensions of the glass powders after UV photolysis of H_2_O_2_.^15^ Each glass powder (15 mg) was placed in a quartz cuvette and suspended in 0.500 mL of a phosphate buffer solution (potassium phosphate buffer, PB, 0.25 M, pH = 7.4) in the presence of DMPO (0.080 M) and H_2_O_2_ (8.0 × 10^–4^ M). The suspension was continuously stirred and irradiated with a 500 W mercury/xenon lamp (Oriel Instruments). The lamp was equipped with a filter with a cutoff at 315 nm in order to facilitate H_2_O_2_ photolysis but prevent the photodegradation of the DMPO molecule. Moreover, an IR water filter was used to avoid the overheating of the suspension during irradiation. After 5, 10, and 30 min of irradiation, a small aliquot of the suspension was collected with a syringe and filtered (CA filter, pore diameter 0.45 μm). A volume of 50 μL of the clear solution was collected using a capillary, and the EPR spectrum was recorded using an X-band EPR spectrometer (Miniscope 100, Magnettech). The same experiment was conducted on a blank solution without glass powder (positive control). All the experiments were repeated at least three times. Results were statistically analyzed using Origin 9 software (OriginLab) by employing the one-way ANOVA test and Tukey’s analysis. Results were considered as significant for *p* <0.05. The specimens’ cytocompatibility was first verified toward human osteoblast progenitor cells (hFOB, ATCC CRL-11372, ATCC, LGC Standards, Milan, Italy) cultivated for 72 h in direct contact with the bioactive glass surface by metabolic activity evaluation (alamarBlue, ThermoScientific, Milan, Italy) and morphology assessment through fluorescence imaging. Then, the scavenging ability for oxygen/nitrogen radicals (RONS) in the presence of cells was evaluated in the supernatants using a specific kit (In vitro ROS/RNS assay, Cell Biolabs, Inc., San Diego, CA, USA), and the specimens’ ability to preserve the cell viability was evaluated after inducing oxidative stress for 72 h by means of H_2_O_2_ (500 mM, 3 h/d) addition into the medium.^22^ Results were statistically analyzed using SPSS v25 software (IBM). Groups were compared using the one-way ANOVA test and Tukey’s posthoc analysis. Results were considered as significant for *p* <0.05.

## Results and Discussion

### Surface Wettability

All the surfaces were hydrophilic (contact angle <90°). In particular, water contact angles of 40° ± 5°, 30° ± 6°, and 65° ± 6° were obtained on SCN 50-35-15, SCN 55-35-10, and SCN 60-35-5, respectively. A high wettability (low contact angle) can be associated with the exposition of −OH groups on the glass surface, as previously reported by the authors.^15,23^ Generally, the lower the contact angle, the higher should be the amount of −OH groups exposed on the glass surface. SCN 60-35-5 presents the lowest wettability according to the expected lower reactivity associated with its high SiO_2_ content. On the other hand, SCN 50-35-15 and SCN 55-35-10 do not follow the expected reactivity scale, since SCN 55-35-10 shows the lowest contact angle, even if it must be mentioned that the difference in the contact angles of these two glasses is limited.

### Zeta Potential Measurements

**Figure 1:**
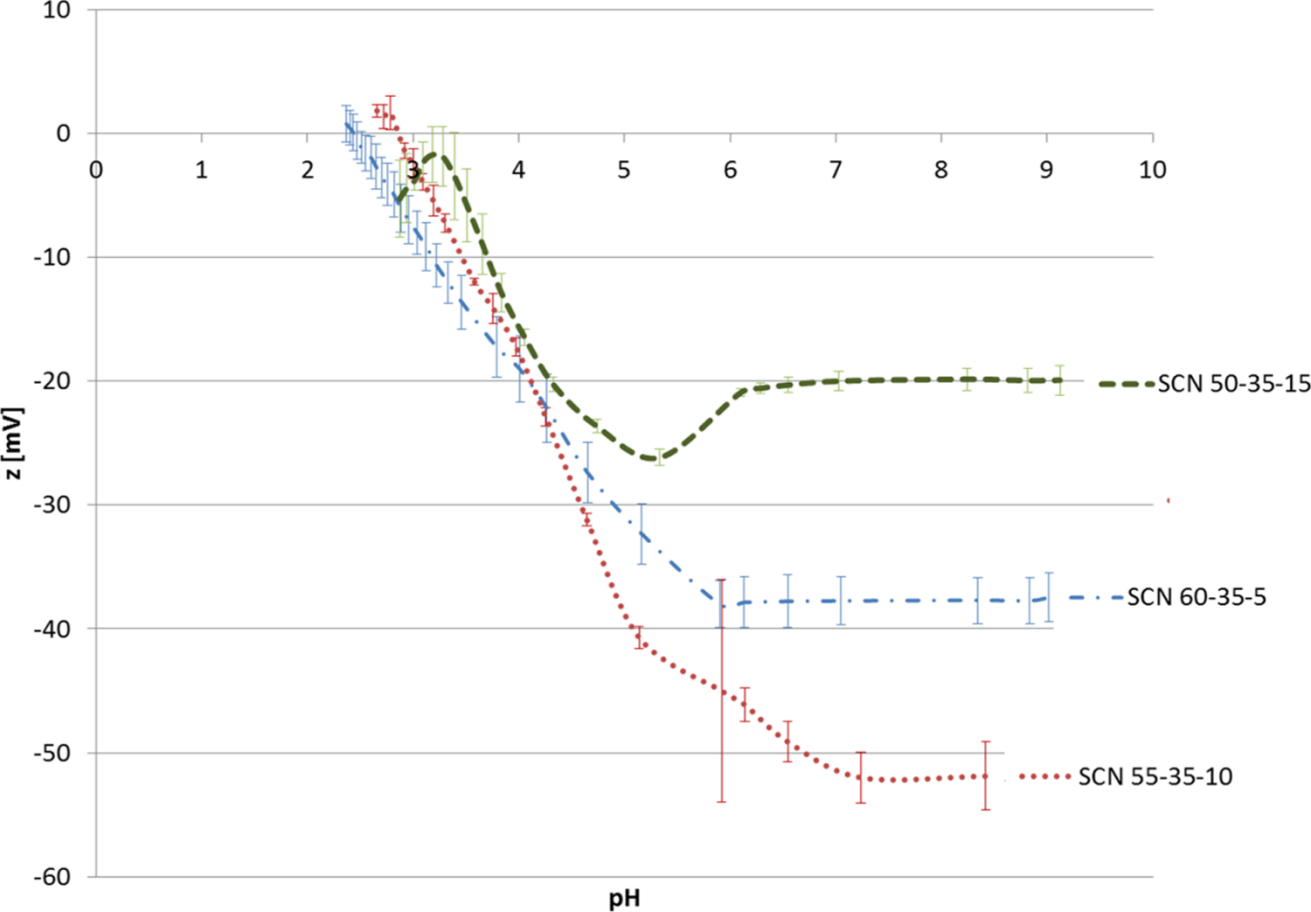
Zeta potential titration curves.

Zeta potential titration curves are shown in Figure 1. The isoelectric point (IEP) for SCN 50-35-15 has not been instrumentally determined because of the reactivity of the sample for pH <3.5, which made the measurement unstable and not reliable; however, it can be extrapolated, giving a value close to 3.2. IEP values of 2.9 and 2.4 have been obtained for SCN 55-35-10 and SCN 60-35-5, respectively. The trend in the IEP values can be associated with the sodium content as an increase in the IEP has been reported with the increase in Na_2_O in the glass composition.^24^ All the glasses have an acidic IEP and consequently a negative surface charge at a physiological pH value; however, the magnitude of this charge is not the same. Values of −20, −50, and −38 mV were obtained for SCN 50-35-15, SCN 55-35-10, and SCN 60-35-5, respectively.

All the curves present a plateau in the basic range, which can be associated with the presence of acidic functional groups (−OH for instance). The onset of the plateau starts at about pH = 6 for all the glasses, but the trend is not similar for all the compositions: SCN 50-35-15 and SCN 60-35-5 show a clear plateau at pH =6, while for SCN 55-35-10, it starts at pH = 6 and stabilizes at values close to 7. This particular trend could be related to a lower acidic strength for −OH groups of this glass^20,25^ or to a higher presence of −OH on its surface compared to SCN 50-35-15 and SCN 60-35-5.

### Surface Chemical Analysis

**Figure 2:**
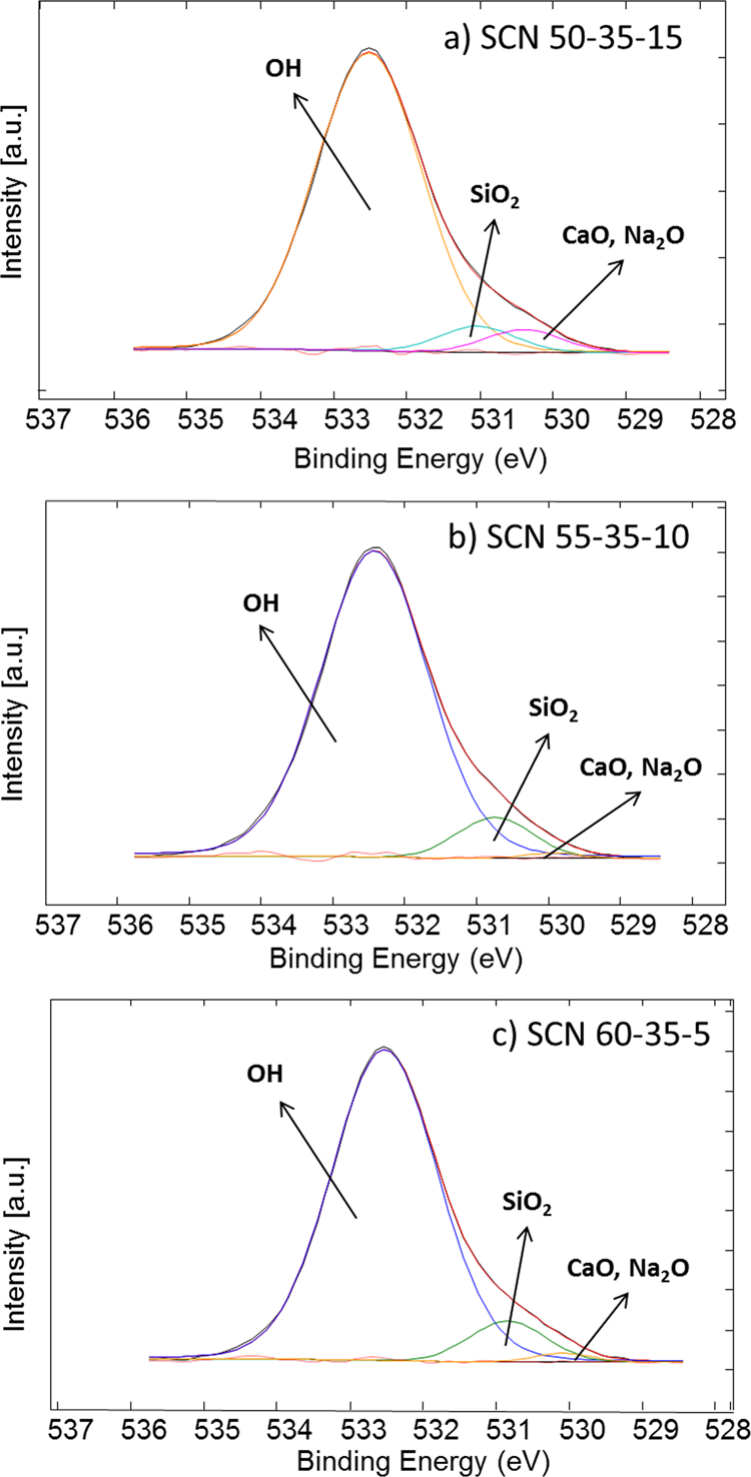
XPS high-resolution spectra (O region).

The high-resolution XPS spectra of the oxygen region are shown in Figure 2. For all the glasses, three main contributions can be detected at about 530, 531, and 532.5 eV, which are attributed to oxides (CaO and Na_2_O), silica, and −OH, respectively, as previously reported.^23^ For all the considered glasses, the −OH signal is the predominant one, evidencing a high hydroxylation degree, which confirms the above reported wettability and zeta potential results. The ratio between the −OH contribution and the other ones (CaO, Na_2_O, and SiO_2_) has been calculated considering the area% of the corresponding peak in the high-resolution XPS spectra of the oxygen region. The ratios obtained are 8.7, 10.5, and 9.7 for SCN 50-35-15, SCN 55-35-10, and SCN 60-35-5, respectively. It can be observed that the trend in hydroxylation degree (XPS) follows the one of the surface charge at physiological pH (zeta potential) and of wettability (contact angle). It can be noticed that the differences detected by XPS are smaller probably because these analyses were performed under high vacuum conditions, and unlike the others, without any contact of the glasses with aqueous media. The most negative surface (SCN 55-35-10) is the one with the highest hydroxylation degree and also the lowest contact angle, suggesting an effect of the hydroxylation degree on both surface charge and wettability.

The high-resolution spectra of the silicon region (not reported) evidence a single contribution centered at 103 eV for all the glasses, which can be associated with Si–O bonds in silica, without significant silica gel formation.^26^ This result is reasonable considering that XPS analyses are performed on glasses without any surface treatment and under dry conditions.

Looking at these results, it can be hypothesized that SCN 60-35-5 has the lowest hydroxylation degree because it is the least reactive one (according to its composition with a high silica content), and SCN 50-35-15, even if it is theoretically the most reactive one (lowest silica content and highest Na_2_O content), presents an intermediate hydroxylation degree probably because of partial −OH condensation (due to high reactivity) more evident in contact with aqueous media. As a consequence, the highest amount of −OH (as evidenced by contact angle, zeta potential, and XPS results) is exposed on the SCN 55-35-10 glass, which is reasonably the glass, in this group, with an intermediate degree of reactivity.

### Bioactivity in SBF

**Figure 3:**
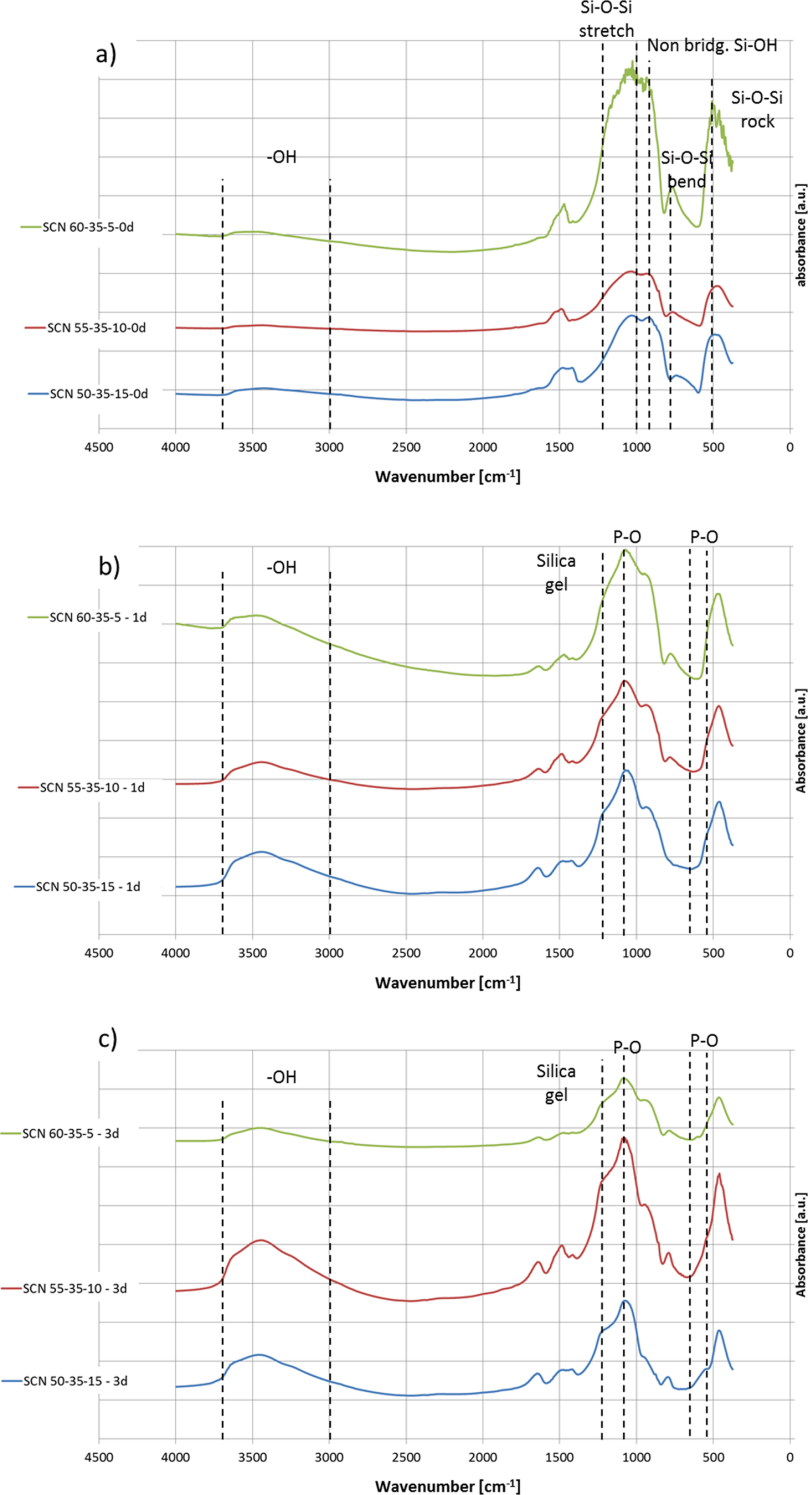
FTIR spectra (a) before soaking, (b) after soaking for 1 day in SBF, and (c) after soaking for 3 days in SBF.

The FTIR spectra of the glasses before and after soaking in SBF (1 and 3 days) are shown in Figure 3.

Before soaking (Figure 3a), all the glasses present signals at about 1000–1200, 780, and 480 cm^–1^ attributable to Si–O–Si stretching, bending, and rocking, respectively. A signal around 930 cm^–1^ can be associated with nonbridging oxygens and Si–OH groups. Finally, the broad band between 3000 and 3600 cm^–1^ can be assigned to −OH groups.^27,28^ All these signals are typical of silica-based bioactive glasses and no significant differences can be highlighted between the different compositions. Considering the penetration depth of FTIR analyses (micron range, not only the outermost surface layer is analyzed as in XPS) and the dry test environment, it is reasonable that the differences in the reactivity among the glasses are not clearly evidenced by this technique.

After soaking in SBF (Figure 3b,c), a significant increase in the band between 3000 and 3600 cm^–1^ can be evidenced, which is associated with the rapid hydration of the glasses upon contact with the solution. No significant differences can be evidenced among the tested glasses in terms of hydroxylation upon contact with SBF up to 3 days. Moreover, a signal around 1200 cm^–1^, attributable to silica gel formation, and one at 1035 cm^–1^, associated with P–O stretching, can be observed on all the glasses from 1 day of soaking (Figure 3b),^28,29^ evidencing the bioactivity of all the tested compositions. A doublet around 600–560 cm^–1^, correlated to P–O stretching,^26,27^ started to appear on SCN 50-35-15 after 3 days of soaking (Figure 3c), confirming its faster hydroxyapatite precipitation. Considering the reactivity of these glasses, it can be inferred that at 1 day of soaking the differences in hydroxylation degree (related to −OH exposition and further condensation) are no more visible because at this time, and more evidently after 3 days, all the glasses show uniform silica gel coverage and the beginning of their phosphate enrichment.

### Radical Scavenging Activity in Inorganic Cell-Free Media

**Figure 4:**
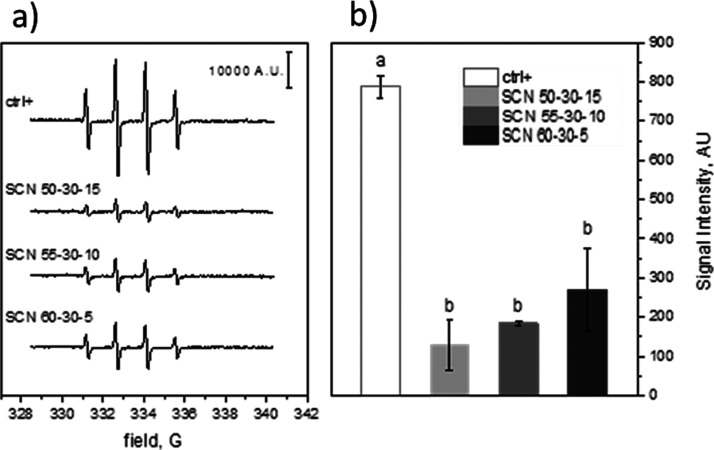
Radical scavenging activity measured by EPR-spin trapping. (a) Representative spectra of [DMPO-OH] and (b) [DMPO-OH] concentration obtained by double integration of EPR spectra of three different determinations. Data are reported as mean ± standard deviation of triplicate experiments. Columns that do not share at least one letter are statistically different (ANOVA, Tukey’s test *p* <0.05).

The radical scavenging activity was measured on sample buffered suspensions (pH = 7.4). As shown in Figure 4a, the EPR spectra recorded with the three samples are compared with the spectrum of a clear solution after 30 min of irradiation. The signal intensity, expressed as A.U., was obtained by double integration of the EPR spectra, and the average intensities (±SD) of three different experiments are shown in Figure 4b. All the samples analyzed (namely, SCN 50-30-15, SCN 55-35-10, and SCN 60-35-5) were able to significantly reduce the concentration of HO**·**, indicating their capability to scavenge free radicals produced by H_2_O_2_ photolysis. This result is in accordance with the literature where the scavenging activity of SiO_2_ toward HO**·** radicals is reported^30^ and as previously observed by the authors for a different silica-based bioactive glass.^15^

The slight trend observed in Figure 4 for the radical scavenging activities, although not of statistical significance, can be ascribed to the different reactivities of the three glasses. It can be hypothesized that SCN 50-35-15 exposes a certain amount of −OH, which is stable in the test time, because Si–OH condensation occurs immediately upon contact with the aqueous medium. On the other hand, SCN 55-35-10 and SCN 60-35-5 have at the beginning a higher amount of exposed −OH (especially SCN 55-35-10) because their tendency to condense is slower, but it could evolve during contact with the aqueous medium in 30 min. Thus, the mechanism through which the tested bioactive glasses were able to decrease the amount of photogenerated HO**·** radicals may be ascribed to the interaction of specific moieties on their surface. Two mechanisms have been proposed: one envisages the abstraction of the hydrogen atom of −OH with production of a siloxyl radical (Si–O**·**) and water and the other is based on the trapping of HO**·** by Si with the formation of a pentacoordinate silicon complex. The direct addition of HO**·** to Si does not seem likely as this element exhibits its highest oxidation state.^30^ Moreover, the role of silanol −OH groups in scavenging HO**·** is corroborated by the evident decrease of such an activity after extensive and largely irreversible dehydroxylation of the silica surface.^31^

### Cytocompatibility and Scavenging Ability for RONS in the Presence of Cells

**Figure 5:**
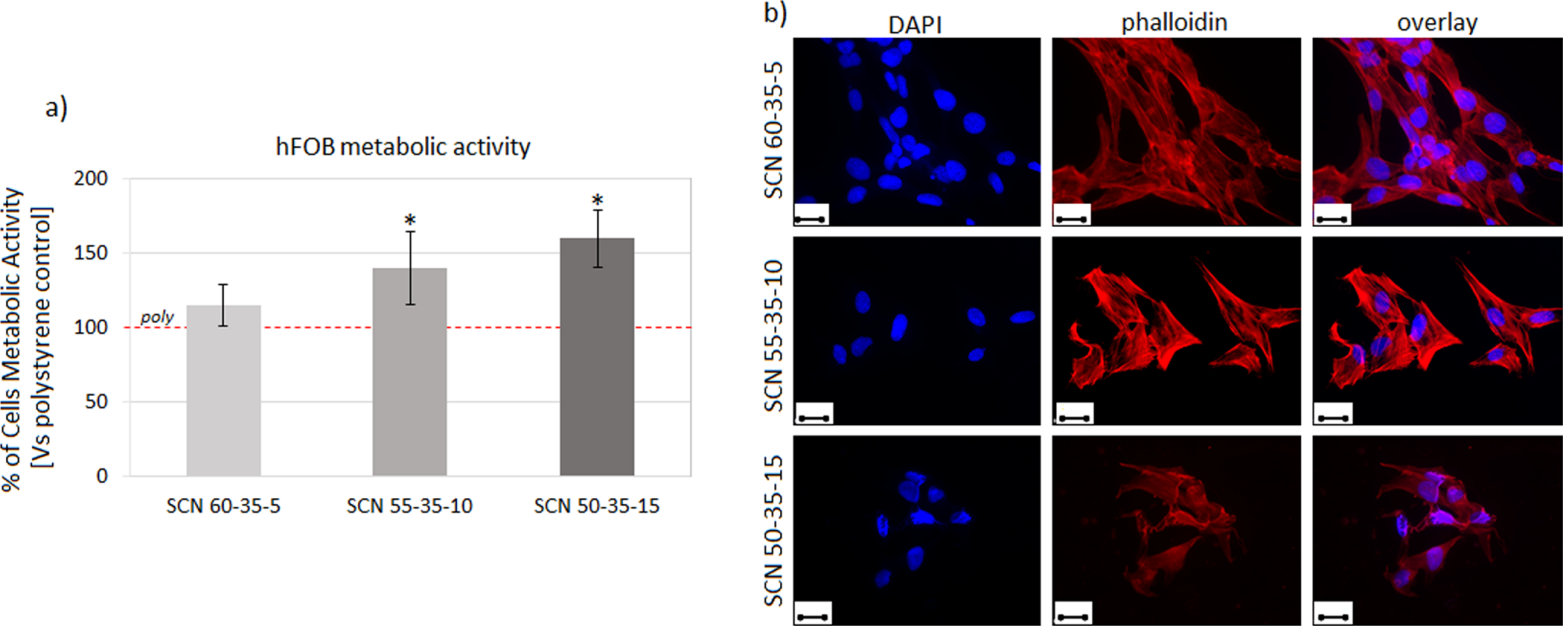
Cytocompatibility evaluation. After 48 h of direct cultivation onto the specimen surface, (a) hFOB metabolic activity was significantly higher for SCN 55-35-10 and SCN 50-35-115 compared to that of the polystyrene control that was considered as 100% (poly, indicated by the red dashed line) thanks to the glass bioactivity (*p* <0.05, indicated by a star). (b) The cell morphology was visually verified by fluorescence imaging, confirming adhesion and spread. Image bar scale = 25 μm.

The specimen cytocompatibility was first evaluated to exclude any possible toxic effects due to the different glass compositions. Accordingly, the gold standard polystyrene was used as a control to normalize the metabolic activity results obtained from the cells cultivated directly onto the specimen surface. hFOB osteoblast progenitor cells were selected as a test model as cells deputed to the self-healing process colonizing the surface of the implantable materials.^29^ Results obtained after 72 h of direct cultivation onto the specimen surface are shown in Figure 5.

Thanks to the bioactivity of glasses, cells displayed a significantly higher metabolic activity when cultivated onto SCN 55-35-10 and SCN 50-35-15 surfaces in comparison to the polystyrene control (poly, indicated by the red dashed line) that was considered as 100% (Figure 5a, *p* <0.05 indicated by a star*). Morphological observation (Figure 5b) based on nuclei (DAPI) and cytoskeleton F-actin filament (phalloidin) staining confirmed that cells properly adhered and spread onto the test specimens.

**Figure 6:**
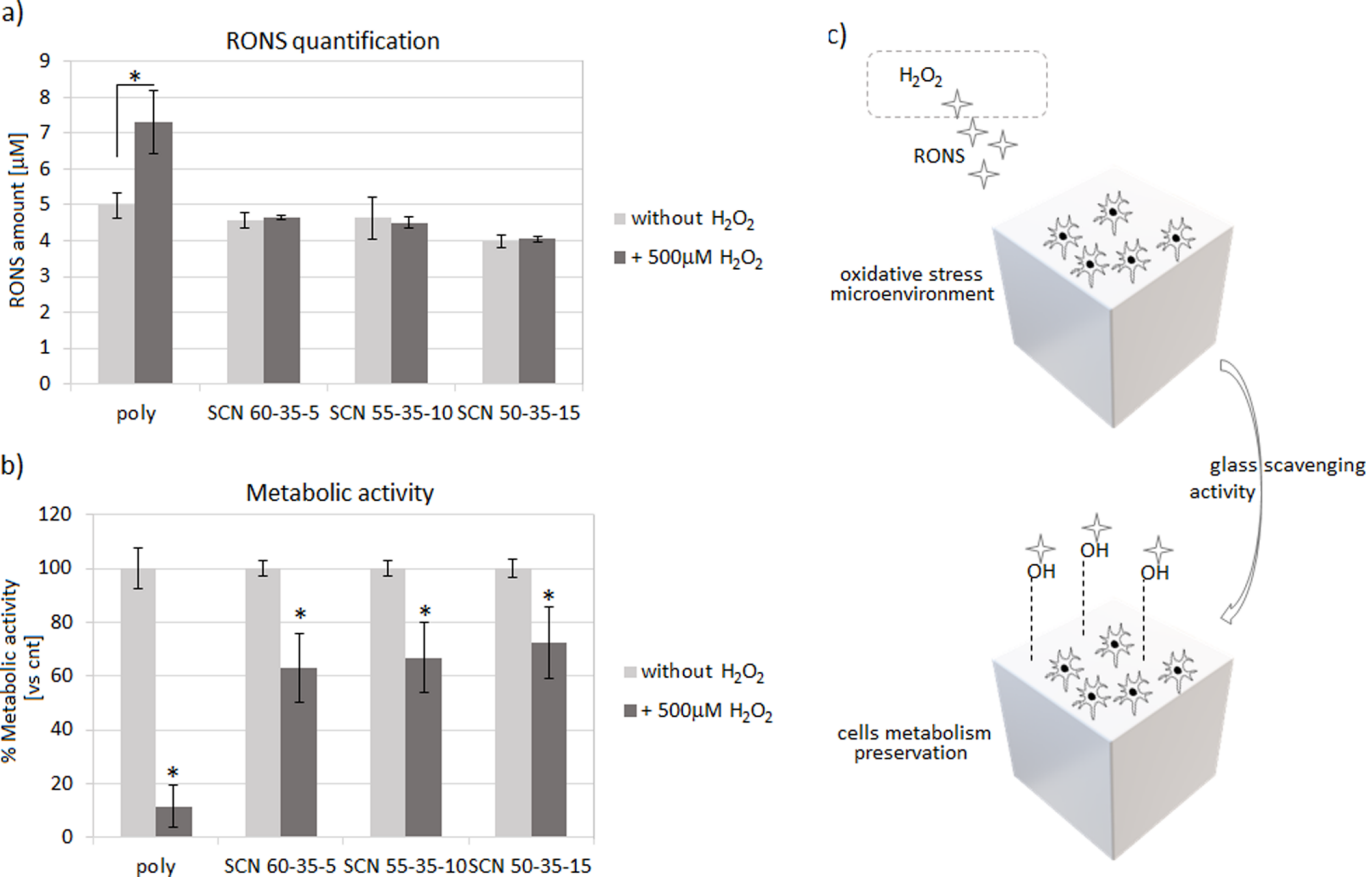
Scavenging activity of specimens. Glasses were able to maintain the released RONS amount upon H_2_O_2_ stress (a) thus preserving >60% of the cells’ metabolism after 72 h of oxidative stress (b). Accordingly, a preventive protective scavenging activity was hypothesized for the glasses in counteracting the side effects due to the RONS production (c).

After confirming the cytocompatibility of bioactive glasses, thus excluding any toxic effect due to the different compositions, experiments were repeated by introducing H_2_O_2_ (500 mM, 3 h/d) into the medium in order to simulate an oxidative stress condition with the aim to test the specimens’ scavenging activity. Accordingly, after 72 h of cultivation in the stressed environment, the RONS amount and the cell metabolic activity were measured and compared to those under the same physiological conditions (i.e., without H_2_O_2_). The results are shown in Figure 6.

As expected, the polystyrene control did not report any ability to reduce RONS increase upon H_2_O_2_ treatment, thus reporting significant results in the active species quantification (Figure 6a, *p* <0.05, indicated by the star). In fact, the overall amount of RONS increased ≈46% (from 4.98 ± 0.34 to 7.33 ± 0.88) upon stimulation, thus showing a fast increase without any contrast.

On the contrary, it can be observed that all the glasses upon H_2_O_2_ stimulation are able to maintain the level of RONS analogous to the ones without stimulation. In detail, the RONS gain included a minimum of ≈1% (SCN-55-35-10, from 4.49 ± 0.59 to 4.63 ± 0.53) and a maximum of ≈3% (SCN-50-35-15, from 4.55 ± 0.21 to 4.75 ± 0.05), therefore reporting in general a negligible increase.

This result evidences the antioxidant activity of this set of bioactive glasses also in the presence of cells. However, no differences among the compositions can be highlighted with this test. This result can be explained considering that after long times of immersion in physiological media (as already revealed after 1 and 3 days in SBF in the abovedescribed FTIR analyses), no significant differences can be highlighted in the hydroxylation degree of the tested materials.

The hypothesis that glasses can act as toxic active species scavengers seems to be confirmed by the cells’ metabolic activity evaluation (Figure 6b). Here, H_2_O_2_ was introduced to turn the environment to a simulated oxidative stress condition for a 72 h long period; then, the metabolic activity of cells was measured and compared to the same values obtained by cells cultivated for 72 h in a basal medium (i.e., without H_2_O_2_) that was considered 100% due to the specimens’ cytocompatibility, as previously shown in Figure 5. Despite all results were significant toward untreated controls (Figure 6b, *p* <0.05 indicated by a star), a noticeable difference was observed between polystyrene (poly) and glasses. In fact, cells cultivated onto polystyrene lost ≈90% of their metabolic activity upon H_2_O_2_ treatment; on the contrary, cells cultivated onto bioactive glasses preserved their viability within a ≈ 60–70% range. Thus, as hypothesized previously, the cells’ metabolic preservation may be ascribed to the exposed −OH groups on the glass surface that were able to scavenge most of the H_2_O_2_-derived RONS, thus avoiding their side effects toward cells (schematized in Figure 6c). Therefore, cell experiments confirmed the scavenging activity of glasses in light of their cellular protection toward toxic active species.

## Conclusions

Three bioactive glasses belonging to the SiO_2_–CaO–Na_2_O system with different SiO_2_ and Na_2_O contents have been designed, synthesized, and characterized in terms of wettability, zeta potential, chemical composition, and bioactivity in order to investigate the role of surface reactivity on the antioxidant behavior. All the glasses showed an acidic IEP, a negative charge at physiological pH, and a high hydroxylation degree. Some differences in composition, surface wettability, and charge at physiological pH have been noticed with a trend related to the degree of hydroxylation, visible in the early stages of their reactivity. All the formulations showed a remarkable radical scavenging activity in cell-free media with a trend correlated to their surface hydroxylation, which is evident at short times of exposition to the environment, and can be related to the different early-stage reactivities of the three glasses. The scavenging ability for RONS has been demonstrated also in the presence of cells for all the formulations. In fact, the ability to limit the RONS increase within a range between 1 and 3% upon stimulation despite the quite different glass reactivities allowed to strongly reduce the active species side effects toward osteoblast progenitors, whose viability was maintained at >60%. Nevertheless, this test did not reveal significant differences among the three compositions. This behavior can be explained considering that upon contact with physiological media for times over 24 h, the set of glasses studied in this work presented a similar hydroxylation degree, which seems to be the key factor for their antioxidant ability.
